# Case Report: SMARCB1-deficient phenotype may be a new specialized type of pleomorphic xanthoastrocytoma associated with poor prognosis

**DOI:** 10.3389/fonc.2025.1527909

**Published:** 2025-03-31

**Authors:** Hui Zhang, Xueping Xiang, Xiaojing Ma, Buyi Zhang, Susu Xu, Xiaojuan He, Jinghong Xu

**Affiliations:** ^1^ Department of Clinical Diagnostic Pathology, The Second Affiliated Hospital of Medical College of Zhejiang University, Hangzhou, China; ^2^ Department of Molecular Pathology, The Second Affiliated Hospital of Medical College of Zhejiang University, Hangzhou, China

**Keywords:** pleomorphic xanthoastrocytoma, SMARCB1, deficiency, DNA methylation profile, genetic characteristics

## Abstract

Pleomorphic xanthoastrocytoma (PXA) is a rare, localized glioma characterized by frequent *BRAF* V600E mutations and *CDKN2A/B* deletions. Compared to *IDH*-wildtype glioblastoma, PXA has a better prognosis. Recently, rare cases of PXA with rhabdoid histological characteristics have been reported, which are titled atypical teratoid/rhabdoid tumor arising in a PXA. However, the genetic characteristics of these cases have rarely been investigated. Herein, we present a 49-year-old woman with a mass in the left frontotemporal region. Microscopically, this mass is composed of the glial and rhabdoid elements, both of which have molecular features of PXA, and the rhabdoid elements assessed using immunohistochemistry for SMARCB1 (INI1) expression demonstrated expression loss. The DNA methylation profile showed a significant calibrated score of 0.81 for methylation class PXA. The tumor was eventually diagnosed as a PXA with SMARCB1 deficiency.

## 1 Introduction

Categorized as circumscribed astrocytic gliomas in the 2021 World Health Organization (WHO) classification of tumors of the central nervous system, 5^th^ edition (CNS WHO), pleomorphic xanthoastrocytoma (PXA) predominantly affects children and young adults. It is characterized by a high frequency of *BRAF* V600E type mutation and homozygous *CDKN2A/B* deletions in molecular pathology (1). PXA is predominantly located in the temporal lobe and grossly appears as a superficial cystic and solid mass. The classic histological features show large pleomorphic, spindle, and lipidized cells, often with numerous eosinophilic granular bodies and reticulin deposition (2, 3). Some rare histological patterns of PXA, such as clear cell patterns (4) and papillary morphologies, have been described in the literature (5).

SMARCB1, also known as integrase interactor 1 (INI1), is ubiquitously expressed in the nuclei of all normal cells and is involved in gene regulation and tumor development (6, 7). It has been revealed that loss of function mutations in this gene result in aggressive rhabdoid tumors. These tumors are known to have common histological and immunohistochemical features such as the appearance of rhabdoid cells (8). Atypical teratoid/rhabdoid tumors (AT/RTs), highly aggressive tumors of the CNS (WHO grade 4) that predominantly affect both infants and children, and are moleculary characterized by a biallelic alteration of the *SMARCB1* (95%) or *SMARCA4* (5%) genes (9, 10). Transcriptome and DNA methylation profiling categorizes AT/RTs into four molecular groups with different methylation levels, designated as: AT/RT-TYR, AT/RT-SHH, AT/RT-MYC, and AT/RT-SMARCA4 (11). To date, six cases of secondary AT/RT arising from PXA have been reported (12–16) (**Table 1**), all of which contained both PXA and rhabdoid tumor components; however, the genetic characteristics of these cases have rarely been explored. To further clarify whether this was a specific type of PXA or secondary AT/RT, we present the case of an adult with morphological features similar to those reported in the literature, with SMARCB1-deficient PXA phenotype.

**Table 1:** Six cases of atypical teratoid/rhabdoid tumor arising in pleomorphic xanthoastrocytoma.

Case	Age/Sex	Location	Tumor composition	Genetic alteration	Follow-up	Author/year	Reference
1	23/M	right frontal	PXA+AT/RT	22q deletion or monosomy 22 BRAF V600E mutation	expire 2 weeks later	Chacko G/2007	(14)
2	8/F	right frontal	PXA+AT/RT	INI1mutation	Not described	Dougherty MJ/2010	(16)
				BRAF V600E mutation			
3	13/F	left cerebellum	PXA+AT/RT	INI1mutation BRAF V600E mutation	expire 5 months later	Jeong JY /2014	(12)
4	23/F	left temporal	PXA+AT/RT	INI1mutation BRAF V600E mutation	expire 8 months later	Uner M /2017	(13)
5	22/F	occipital	aPXA+AT/RT	INI1mutation BRAF V600E mutation	expire 5 months later	Nobusawa S/2019	(15)
6	27/F	left parahippocampal	PXA+AT/RT	INI1mutation BRAF V600E mutation CDKN2A deletion	expire 5 months later	Nobusawa S/2019	(15)
7	49/F	left frontotemporal	aPXA+AT/RT	INI1mutation BRAF V600E mutation CDKN2A deletion	expire 2 months later	The present case	

aPXA, anaplastic pleomorphic xanthoastrocytoma; AT/RT, Atypical teratoid/rhabdoid tumors.

## 2 Case presentation

**Figure 1:**
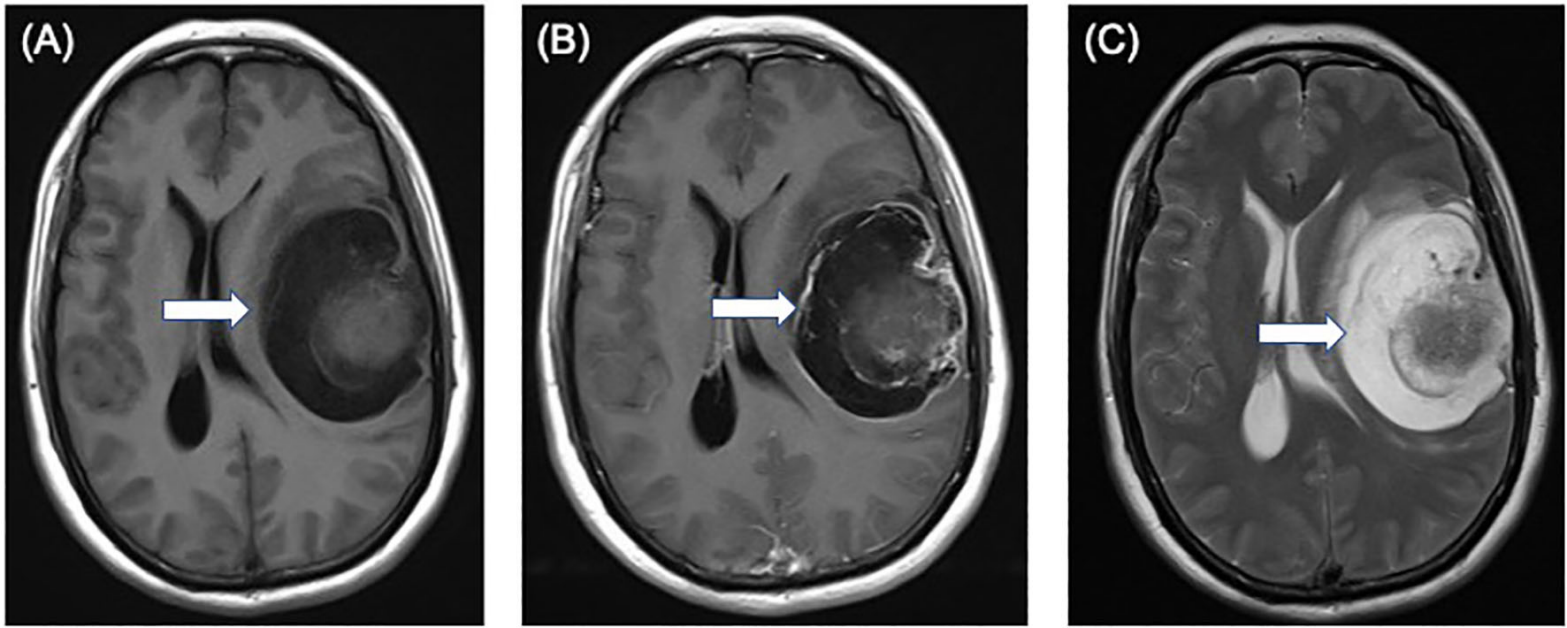
Preoperative magnetic resonance images showing a 7.1 × 5.8 × 5.0 cm solid-cystic mass in the left frontotemporal region. **(A)** T1-weighted axial image shows an isointense nodule (arrow) of the cystic wall located on the surface side of the brain. **(B)** the cystic wall shows obviously enhanced and the nodule of the cystic wall shows slightly and not uniformly enhanced (arrow) after gadolinium contrast injection. **(C)** T2-weighted axial image shows an isointense nodule (arrow) of the cystic wall with hyperintense cystic area.

A 49-year-old female patient presented with sudden onset of slurred speech and right-sided limb weakness 20 days ago without any obvious cause, accompanied by headache but no nausea or vomiting. Magnetic resonance imaging of the brain displayed a 7.1 × 5.8 × 5.0 cm mass in the left frontotemporal region, T1-weighted axial image shows an isointense nodule of the cystic wall located on the surface side of the brain (**Figure 1A**). The cystic wall shows obviously enhanced and the nodule of the cystic wall shows slightly and not uniformly enhanced after gadolinium contrast injection (**Figure 1B**). T2-weighted axial image shows an isointense nodule of the cystic wall with hyperintense cystic area (**Figure 1C**).

The patient underwent a craniotomy with total tumor excision. The tumor was located at the top of the frontoparietal region, exhibiting heterogeneous qualities during surgery, and was highly vascularized with mucoid changes at the base. The boundary between the tumor and surrounding brain tissue was clearly observed. The surgeon removed the tumor as much as possible along the tumor boundary.

**Figure 2:**
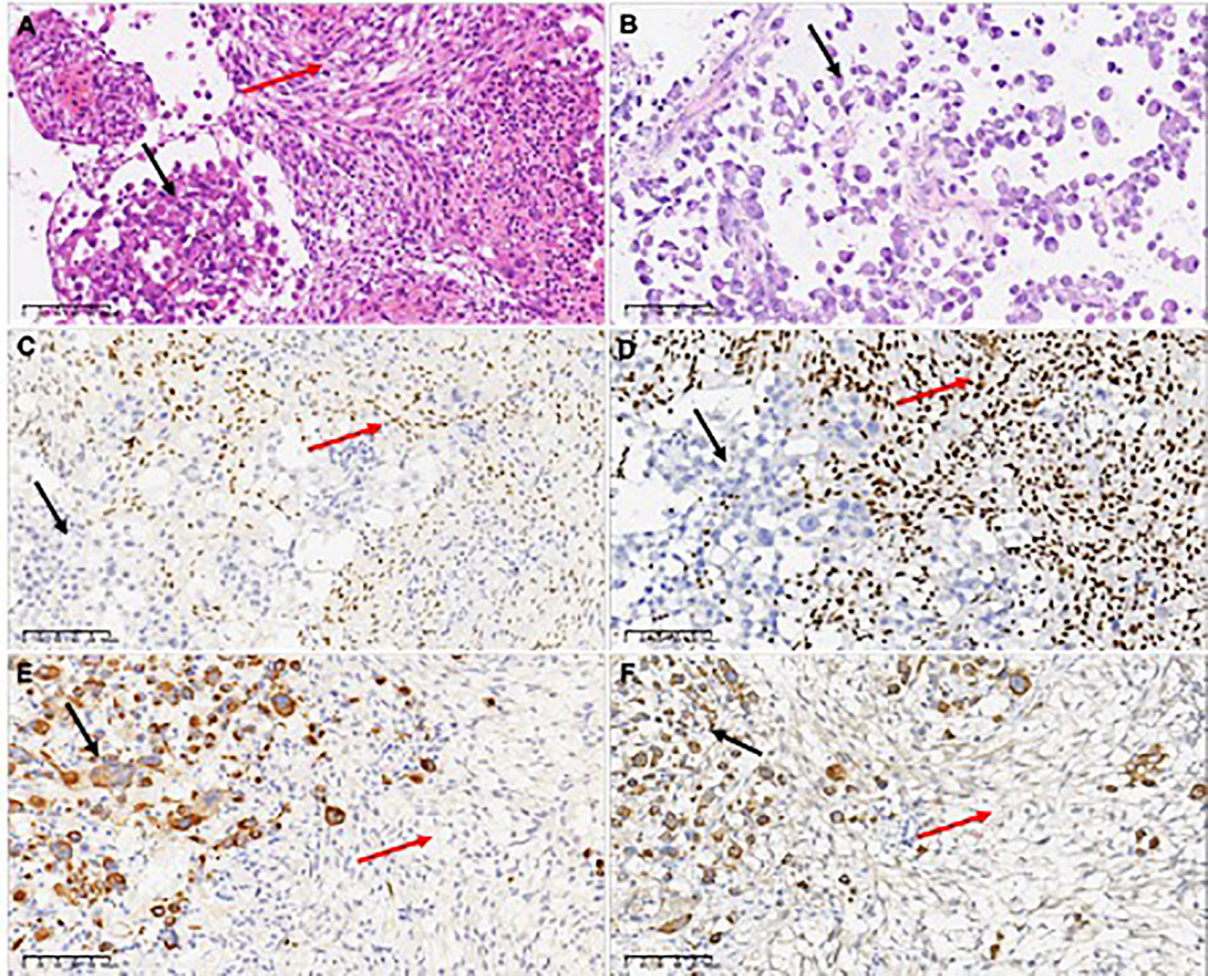
H&E-stained the first surgical sample sections revealed a tumor with two distinct spindle and rhabdoid morphological features **(A)**, while the second surgical sample showed only rhabdoid tumor cells **(B)**. Only spindle cells expressed the glial differentiation marker Olig2 **(C)**. INI1 expression was different between the two components, which was retained in the spindle element and lost in the rhabdoid element **(D)**. Epithelial-derived marker CK(AE1/AE3) **(E)** and myogenic marker Myoglobin **(F)** were observed to be expressed in rhabdomyoid cells, but not in spindle cells. In the figure, the black arrows refer to rhabdoid components, and the red arrows refer to spindle components.

Microscopic examination revealed a tumor with two distinct spindle and rhabdoid morphological features (**Figure 2A**). The rhabdoid cells component are characterized by discernible nucleoli, eccentric nuclei, and prominent eosinophilic cytoplasm. Tumors showed distinct signs of malignancy, including brisk mitotic activity and geographic necrosis. The abundant cytoplasmic vacuoles suggested xanthomatous changes in the spindle areas, and mitotic figures of 6–7 mitoses/10 High Power Fields (HPFs) in the spindle cell region.

Immunohistochemical staining revealed a clear contrast between the two components. GFAP and Olig-2 were immunoreactive only in the spindle elements (**Figure 2C**). Spindle cells retained nuclear expression of INI1, whereas rhabdoid cells showed complete loss of INI1 expression (**Figure 2D**). The Ki-67 proliferation index was lower in spindle cells (10%) than in the rhabdoid component (70%), indicating a markedly elevated proliferation index in the later. CK (AE1/AE3) (**Figure 2D**), myoglobin (**Figure 2E**), epithelial membrane antigen, and p53 proteins were detected only in the rhabdoid component. Both desmin and CD34 were negative for both elements, and both components tested positive for vimentin and BRAF V600E.These results indicate that spindle cells and rhabdoid cells have distinct morphological characteristics and immunophenotypes.

**Figure 3:**
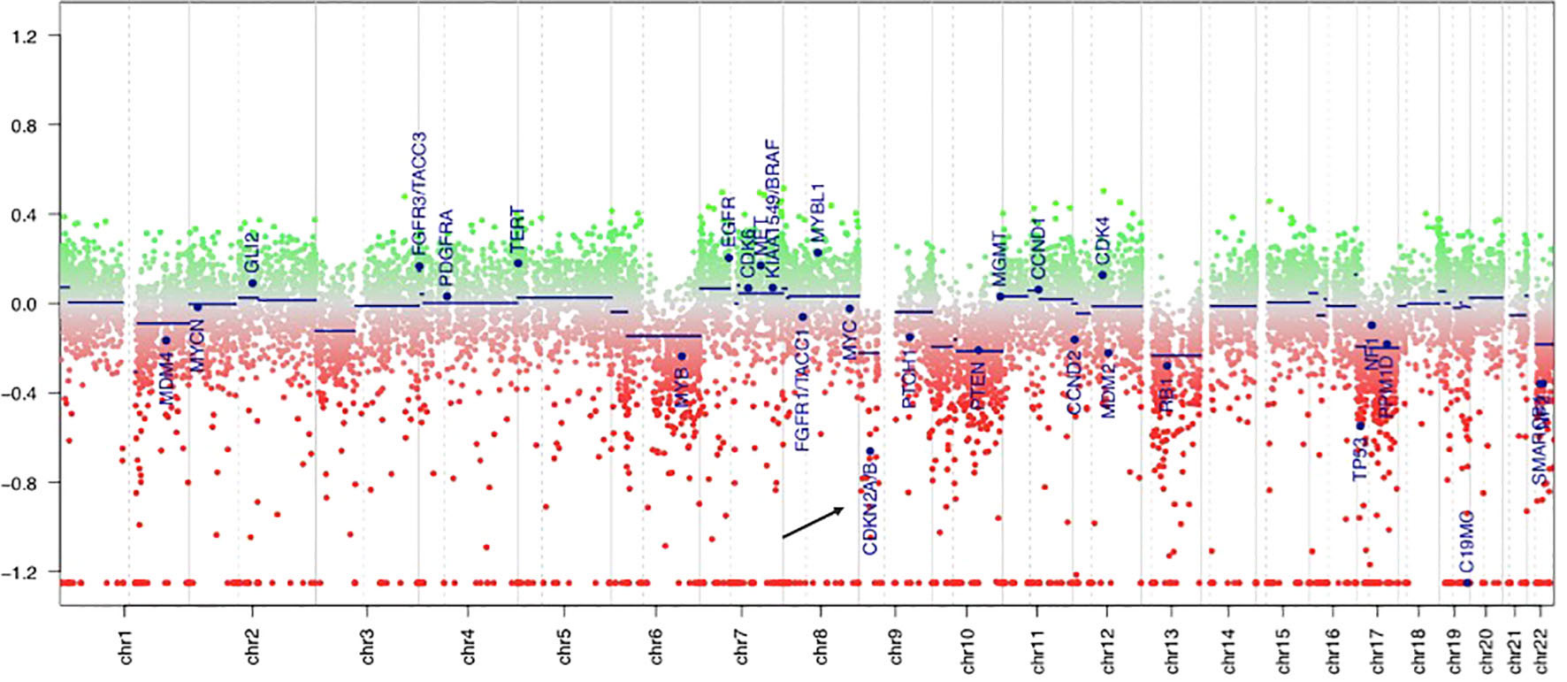
DNA methylation-derived copy number variation showed the deletion of *CDKN2A* gene (the black arrow), which is located in the short arm of chromosome 9. The green area indicates copy number gain in chromosome, and the red area indicates copy number loss. Individual copy number profiles were calculated based on raw methylated/unmethylated signals using conumee package in “R Studio”.

PCR analyses of the first tumor revealed a *BRAF V600E* mutation, and fluorescence *in situ* hybridization demonstrated homozygous *CDKN2A* deletion in both components. The molecular markers of glioblastoma were assessed, and all results were negative: the *IDH* and *TERT* promoter regions were wild-type, *EGFR* was not overexpressed, and the +7/-10 chromosome copy number was unchanged. The DNA methylation profile generated using the Infinium MethylationEPIC v2.0 BeadChip array (Illumina, San Diego, CA, USA) demonstrated a significantly calibrated score of 0.81 for the PXA methylation class in the brain tumor classifier v12.5. Additionally, the *MGMT* promoter was unmethylated, and a genome-wide copy number analysis revealed homozygous *CDKN2A/B* deletions (**Figure 3**).

Two weeks after the first surgery, a cranial Computed Tomography (CT) scan indicated the possibility of postoperative edema and fluid accumulation around the surgical area, prompting the decision to perform a second surgery. The original tumor bed was found to be overgrown with tumor tissue during the second operation. Microscopic examination of the second tumor revealed a purely rhabdoid cell morphology without any spindle cell components (**Figure 2B**). Molecular analysis showed that the tumor tissue, composed solely of rhabdoid cells, also harbored a *BRAF V600E* mutation and homozygous *CDKN2A* deletion, molecular signatures of PXA, along with loss of INI1 expression. The patient died two months postoperatively.

## 3 Discussion

All reported cases exhibited classic histological features of PXA and rhabdoid components, accompanied by loss of INI1 expression (12–15). The authors proposed that these cases represented PXA secondary to AT/RT. However, due to limitations in diagnostic techniques at the time, the reported cases did not undergo comprehensive molecular testing. In our case, the histomorphology was similar to that of the reported cases, with both spindle cell and rhabdoid components demonstrating molecular features of PXA. However, immunohistochemistry revealed completely distinct immunophenotypes between the two components, and DNA methylation profiling clustered the tumor within the PXA group. We therefore consider this case to be PXA with loss of INI1 expression rather than secondary AT/RT.

In recent years, Thomas et al. reported three cases of AT/RT with molecular features of PXA (17), and Dottermusch also reported a similar case in an elderly patient (20). Interestingly, in Thomas’s study, two patients had a history of PXA, and the recurrent tumors exhibited purely rhabdoid morphology. The authors suggested that this might be due to sampling limitations or the rapid growth of the highly malignant rhabdoid component, which could overshadow the PXA component in recurrent tumors. In all reported cases, including ours, methylation profiling consistently clustered within the PXA group. While in reports of AT/RTs secondary to other neuroepithelial tumors, DNA methylation profiling confirmed that these secondary AT/RTs were clustered into the AT/RT-MYC group (18). Therefore, AT/RT-MYC may be the predominant subgroup for secondary RT, consistent with the frequent occurrence of homozygous *INI1* deletions and the older age of patients with secondary AT/RT.

In our case of SMARCB1-deficient PXA, the nodule of the cystic wall showed slightly and not uniformly enhanced, which maybe a distinguished feature of SMARCB1-deficient PXA from the other PXA or indicate the malignancy of the tumor on imaging. Imaging techniques may also be used to provide a distinction between typical and SMARCB1-deficient PXA (19).

Summarizing all reported cases, PXA with INI1 loss is characterized by rapid progression, high malignancy, and poor prognosis (12–15). In the present case, the patient died two months after the first surgery. This indicates that PXA with a SMARCB1-deficiency exhibits highly-malignant biological behavior, may represent a distinct and unique subtype of PXA. As the number of reported cases of SMARCB1-deficient PXA is limited, the molecular characteristics and comprehensive epigenetics of this entity remain poorly studied, and more cases are needed to fully characterized SMARCB1-deficient PXA.

In conclusion, we present an adult case of SMARCB1-deficient PXA characterized by the focal loss of INI1 expression harboring a *BRAF V600E* mutation and *CDKN2A* homozygous deletion with two distinct morphological and immunohistochemical features. Through molecular analysis and literature review, concomitant SMARCB1 deficiency is suggested to be a special type of PXA with poor clinical prognosis. Further clinicopathological and genetic analyses of additional cases are necessary for better tumor characterization.

## Data Availability

The datasets presented in this article are not readily available because of ethical and privacy restrictions. Requests to access the datasets should be directed to the corresponding author.
